# Chemokine Receptor CCR8 Is Required for Lipopolysaccharide-Triggered Cytokine Production in Mouse Peritoneal Macrophages

**DOI:** 10.1371/journal.pone.0094445

**Published:** 2014-04-08

**Authors:** Tomoyuki Oshio, Rei Kawashima, Yuki I. Kawamura, Teruki Hagiwara, Noriko Mizutani, Toshihiko Okada, Takeshi Otsubo, Kyoko Inagaki-Ohara, Akihiro Matsukawa, Tatsuya Haga, Shigeru Kakuta, Yoichiro Iwakura, Seijiro Hosokawa, Taeko Dohi

**Affiliations:** 1 Department of Gastroenterology, Research Center for Hepatitis and Immunology, Research Institute, National Center for Global Health and Medicine, Ichikawa, Chiba, Japan; 2 Department of Dermatology, Jichi Medical University, Shimotsuke, Tochigi, Japan; 3 Kitasato University Graduate School of Medical Sciences, Sagamihara, Kanagawa, Japan; 4 Department of Pathology & Experimental Medicine, Okayama University, Okayama, Okayama, Japan; 5 Department of Life Science, Gakushuin University, Mejiro-ku, Tokyo, Japan; 6 Department of Biomedical Science, Graduate School of Agricultural and Life Sciences, The University of Tokyo, Bunkyo-ku, Tokyo, Japan; 7 Division of Experimental Animal Immunology, Research Institute for Biomedical Sciences, Tokyo University of Science, Noda, Chiba, Japan; 8 Department of Applied Chemistry, Faculty of Advanced Science and Engineering, Waseda University, Shinjuku-ku, Tokyo, Japan; National Institute of Infectious Diseases, Japan

## Abstract

Chemokine (C-C motif) receptor 8 (CCR8), the chemokine receptor for chemokine (C-C motif) ligand 1 (CCL1), is expressed in T-helper type-2 lymphocytes and peritoneal macrophages (PMφ) and is involved in various pathological conditions, including peritoneal adhesions. However, the role of CCR8 in inflammatory responses is not fully elucidated. To investigate the function of CCR8 in macrophages, we compared cytokine secretion from mouse PMφ or bone marrow-derived macrophages (BMMφ) stimulated with various Toll-like receptor (TLR) ligands in CCR8 deficient (*CCR8^-^* ^/-^) and wild-type (WT) mice. We found that *CCR8^-/-^* PMφ demonstrated attenuated secretion of tumor necrosis factor (TNF)-α, interleukin (IL)-6, and IL-10 when stimulated with lipopolysaccharide (LPS). In particular, LPS-induced IL-10 production absolutely required CCR8. CCR8-dependent cytokine secretion was characteristic of PMφ but not BMMφ. To further investigate this result, we selected the small molecule compound R243 from a library of compounds with CCR8-antagonistic effects on CCL1-induced Ca^2+^ flux and CCL1-driven PMφ aggregation. Similar to *CCR8^-/-^* PMφ, R243 attenuated secretion of TNF-α, IL-6, and most strikingly IL-10 from WT PMφ, but not BMMφ. *CCR8^-/-^* PMφ and R243-treated WT PMφ both showed suppressed c-jun N-terminal kinase activity and nuclear factor-κB signaling after LPS treatment when compared with WT PMφ. A c-Jun signaling pathway inhibitor also produced an inhibitory effect on LPS-induced cytokine secretion that was similar to that of CCR8 deficiency or R243 treatment. As seen in *CCR8^-/-^* mice, administration of R243 attenuated peritoneal adhesions *in vivo*. R243 also prevented hapten-induced colitis. These results are indicative of cross talk between signaling pathways downstream of CCR8 and TLR-4 that induces cytokine production by PMφ. Through use of *CCR8^-/-^* mice and the new CCR8 inhibitor, R243, we identified a novel macrophage innate immune response pathway that involves a chemokine receptor.

## Introduction

Chemokines are small proteins with a molecular mass of 6–14 kDa that induce chemotaxis by binding to G-protein-coupled receptors (GPCRs) on the cell surface [1], [2]. One member of the C–C motif chemokine superfamily, CCL1/I-309, binds to the chemokine receptor CCR8 and induces Ca^2+^ influx and monocyte migration in humans [3]. The ligand of mouse CCR8 was identified as T cell activation-specific gene 3 (TCA3)/CCL1 [4], and mouse CCL8 was recently reported to be a second agonist for mouse CCR8 [5], [6]. CCL1 is a chemoattractant of natural killer (NK) cells, monocytes/macrophages, neutrophils, and regulatory T cells [7]–[9]. It has been reported that CCR8 is the predominant chemokine receptor expressed in T helper type 2 (Th2) cells [10], [11]. The CCL1/CCL8-CCR8 system is involved in the pathology of various inflammatory diseases. For examples, CCL1 is upregulated in Th2-dominant diseases such as asthma and atopic dermatitis [12], [13]. In a mouse model of ovalbumin (OVA)-induced atopic dermatitis, CCL8 was shown to be highly expressed in the skin, where it induces the migration of a population of CCR8-positive IL-5-enriched Th2 cells into the skin, thereby driving eosinophilic inflammation. In addition to these reports of CCR8-positive T cells, CCR8-expressing macrophages also play significant roles in several pathological situations. For example, CCL1 and CCR8 mediate postoperative peritoneal adhesion development in mice [14], CCL1 is produced by mesothelial cells and macrophages in the peritoneal cavity and is a potent enhancer of CCR8 expression in peritoneal macrophages (PMφ) [14], and PMφ produce CCL1 upon inflammatory stimulation. The CCL1/CCR8 pathway activates itself through a positive autocrine/paracrine loop in the peritoneal cavity. *In vitro* stimulation of the PMφ with CCL1 on mesothelial cell layer leads to macrophage aggregation. In mice, such CCR8-positive macrophage aggregates are seen *in vivo* at the serosal sites of peritoneal adhesions induced by acute colitis or surgical manipulation of the peritoneal cavity. Adhesions are efficiently prevented by anti-CCL1 antibody or by *CCR8* gene deficiency in mouse models [14]. Although CCL1 is not the primary chemokine secreted into the peritoneal cavity during laparotomy in humans [15], inflammatory macrophages in lung tissue from patients with chronic obstructive pulmonary disease (COPD) express high levels of CCR8. In COPD, potential interaction with Toll-like receptor (TLR)-4 was suggested because CCL1 induces superoxide and proinflammatory cytokine release from macrophages in the presence of lipopolysaccharide (LPS) [16]. A type 1 diabetes model demonstrated that CCL1 produced by diabetogenic CD4^+^ T cells mediates recruitment of large numbers of CCR5-, CXCR3-, and CCR8-expressing macrophages into the pancreas [17]. The involvement of CCR8 in these diseases suggests that it plays a role in inflammatory/allergic responses by inducing tissue damage and remodeling. Therefore, blockade of CCR8 may be beneficial in alleviating or preventing inflammatory events. Indeed, attempts to identify pharmacological antagonists of CCR8 have been made [18]–[20].

Using mice deficient in the *CCR8* gene (*CCR8^-/^* ^-^), we investigated the role of CCR8 in macrophages activated by inflammatory stimuli. We report here for the first time that LPS-triggered cytokine production by macrophages depends largely on CCR8. The small molecule, R243, antagonized the effect of CCL1-CCR8 *in vitro* and demonstrated potent anti-inflammatory effects in peritoneal adhesions and colitis models *in vivo*. Through a mechanistic study of the effects of CCR8 deficiency and R243 treatment, we identified a new pathway involving cross talk between the TLR4 and CCR8 signaling pathways.

## Materials and Methods

### Ethics Statement

All experiments using mice were performed according to the Institutional Guidelines for the Care and Use of Laboratory Animals in Research with the approval of the Animal Care and Use Committee of the Research Institute, National Center for Global Health and Medicine (13064 and 13066). Animals were anesthetized with ketamine and xylazine for all surgeries and intrarectal injections, and every effort was made to minimize suffering.

### Mice

Male, 8–10-week-old C57BL/6J mice were obtained from CLEA Japan. *CCR8^-/-^* mice from the C57BL/6 strain were originally generated at The Institute of Medial Science, The University of Tokyo (Yabe R. *et al*., submitted). Mice were maintained under pathogen-free conditions at the Research Institute, National Center for Global Health and Medicine.

### Cell culture and stimulation

Peritoneal exudate cells were collected from naïve mice using endotoxin-free phosphate-buffered saline (PBS) and were incubated in RPMI1640 medium with 1% fetal bovine serum (FBS) and penicillin/streptomycin in a 96-well plate for 45 min (2×10^5^ cells/well). After removal of nonadherent cells, adherent cells were used as PMφ. To obtain BMMφ, mice were sacrificed, and the femoral marrow was flushed with PBS. Cells were then resuspended in Dulbecco's Modified Eagle Medium (DMEM) with 10% FBS and 10 ng/mL of macrophage colony-stimulating factor and incubated for 7 days in a 10-cm culture dish. Differentiated macrophages were harvested with a silicon rubber scraper and then washed with PBS and cultured in a 96-well plate (2×10^5^ cells/well). PMφ and BMMφ were stimulated with TLR ligands in RPMI1640 medium with 1% FBS for 24 h, unless otherwise indicated, and/or treated with small molecule compound R243 at the indicated concentrations or with 0.1% dimethylsulfoxide (DMSO) as a vehicle. The culture supernatant was used in cytokine assays. For some experiments, mouse PMφ were stimulated with LPS for 24 h in the presence of signal inhibitors or antibodies to mouse CCL1 or CCL8.

### Small molecule compounds

We selected R243 from a library of compounds (PharmaGCHEM) purchased from PharmaDesign, Inc., Tokyo, Japan. This library consists of 1000 compounds, which were selected based on predicted inhibition of the chemokine receptors CCR1, CCR2, CCR3, CCR4, CCR5, CCR8, and CX3CR1. We prepared mouse CCR8-DsRed-expressing Chinese hamster ovary (CHO) cells and stimulated them with 50 ng/mL of CCL1. A Ca^2+^ influx assay was performed in 96-well-plates using a FLIPR CA 4 Assay Kit (Molecular Devices Japan, Tokyo, Japan) and a microplate reader Flexstation 3 (Molecular Devices). R243 was selected for its inhibition of CCL1-induced Ca^2+^ flux. For *in vitro* and *in vivo* experiments, R243 was purchased from Zelinsky Institute Inc. (Newark, DE). For some experiments, R243 was newly synthesized in the laboratory at Waseda University.

### Chemokine-induced macrophages aggregation (CIMA) assay

The CIMA assay was established as described previously [14]. Briefly, mouse mesothelial cells were cultured in a 24-well dish until confluent. Naïve mouse PMφ were added to this culture and incubated with CCL1 (5 ng/mL) with or without R243 for 24 h at 37°C. The formation of cell aggregates was quantified as the aggregation area by capturing an image with a BX50 microscope (Olympus, Tokyo, Japan) equipped with a charged-couple device (CCD) camera. Images were analyzed using NIH ImageJ 1.46R software (National Institutes of Health, Bethesda, MD).

### Peritoneal adhesion and mouse models of colitis

Three types of experiments were performed after laparotomy, as described in the Method S1, for *in vivo* models of postoperative peritoneal adhesions. Colitis was induced by intrarectal administration of a 2% solution of 2,4,6-trinitrobenzenesulfonic acid (TNBS) in PBS:ethanol (1∶1). To induce acute inflammatory responses, TNBS was administered on days 0 and 2 at 70 μg/g body weight, and colon tissues were obtained on day 4. R243 in PBS with 0.2% mouse serum was injected intraperitoneally at a concentration of 0.04 mg/mL (0.2 mg/kg dose) 1 h before TNBS injection or immediately after surgery on day 0. Control mice were injected with 0.4% DMSO in PBS with 0.2% normal mouse serum. The scoring systems used for the *in vivo* models are described in the Method S1.

### Statistical analysis

Data were statistically analyzed using Prism 4 software (GraphPad Software, Inc) with the method indicated in the legend of each figure. Results were considered statistically significance at P<0.05.

Additional information regarding reagents, cytokine enzyme-linked immunosorbent assay (ELISA), quantitative RT-PCR, flow cytometory, cell viability, chemotaxis, immunofluorescence tests, phosphoprotein assays, *in vivo* models, and scoring systems are provided in the Supporting Information (Method S1).

## Results

### CCR8 deficiency reduces cytokine secretion by mouse PMφ and BMMφ

**Figure 1:**
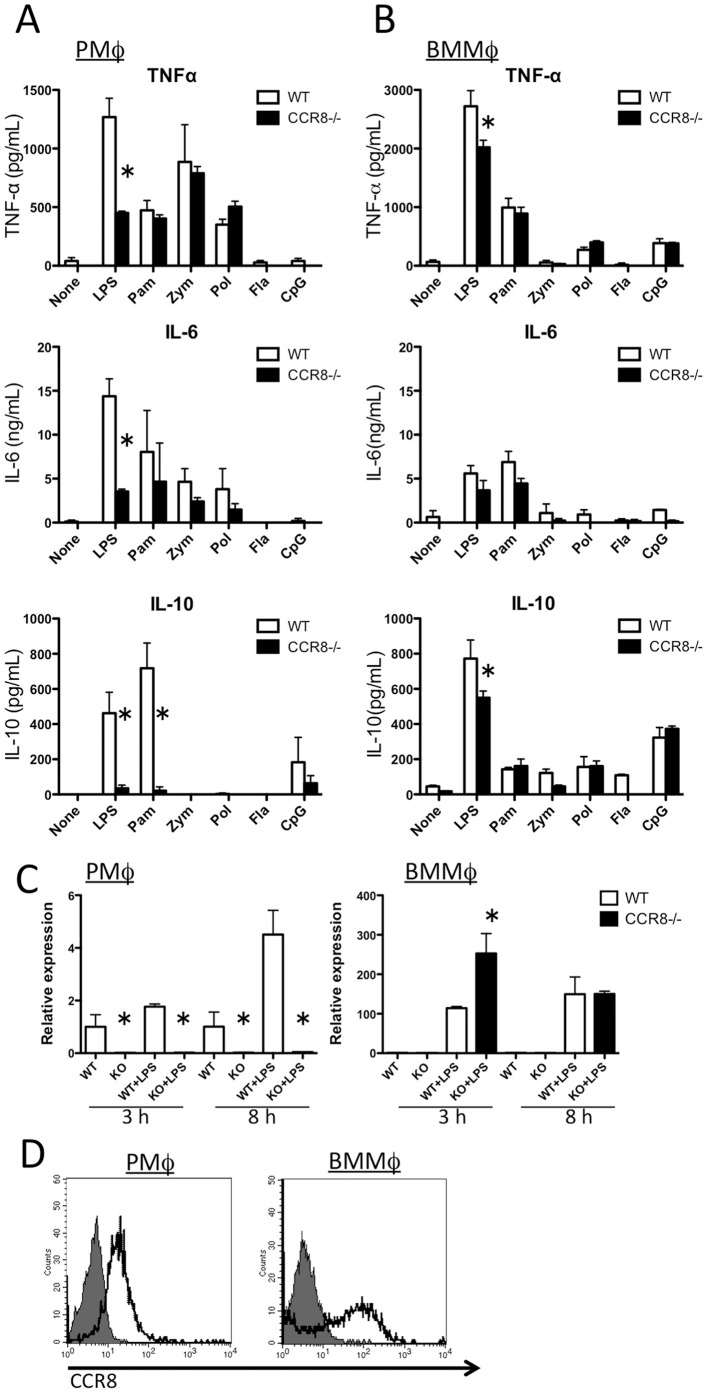
LPS-induced cytokine production by mouse PMφ is dependent on CCR8. (A) PMφ or (B) BMMφ collected from naïve WT or *CCR8^-/-^* mice were stimulated with LPS (100 ng/mL) Pam3CSK4 (Pam, 100 ng/mL), zymosan (Zym, 1 μg/mL), PolyI:C (Pol, 100 μg/mL), flagellin (Fla, 1 μg/mL), or CpG-ODN (CpG, 1 μg/mL) for 24 h. The levels of cytokines in the culture medium were measured. (C) PMφ or BMMφ prepared from WT or CCR8^-/-^ mice was stimulated with LPS as above for 3 or 8 hours, and mRNA levels for IL-10 was measured with quantitative RT-PCR. Values were normalized using 18 s rRNA and shown as relative expression levels to WT cells without LPS of each time point. In (A–C), data are shown as mean plus a standard deviation (SD) determined from triplicate assays for each condition. *Statistically significant difference between WT and *CCR8^-/-^* cells (unpaired two-tailed t test). (D) PMφ or BMMφ gated as F4/80^+^ cells were obtained from naïve WT mice and stained with anti-CCR8 antibody (solid line) or without antibody (shaded histogram) analyzed with flow cytometory.

Based on our previous result that CCL1/CCR8 is involved in the innate immune responses in the peritoneal cavity [14], we examined the effect of CCR8 deficiency in macrophage responses to various TLR ligands. PMφ produced TNF-α and IL-6 in response to LPS (TLR4 ligand), Pam3CSK4 (ligand for TLR1 and TLR2), zymosan (TLR2 ligand), and poly I:C (TLR3 ligand), but not in response to flagellin (TLR5 ligand) or CpG-ODN (TLR9 ligand) (Fig. 1A). We found that LPS-induced TNF-α and IL-6 production, but not other TLR ligands, was clearly attenuated in *CCR8^-/-^* PMφto levels less than 50% of that in WT PMφ. LPS and Pam3CSK4 strongly induced IL-10 production in WT PMφ and almost completely suppressed IL-10 production in *CCR8^-/-^* PMφ. On the other hand, CCR8 deficiency did not affect the levels of any of these cytokines produced by BMMφ when compared with that from PMφ, although moderate inhibition was observed for LPS-stimulated BMMφ secretion of TNF-α and IL-10 (Fig. 1B). Reduced secretion of IL-10 was also confirmed at transcriptional level (Fig. 1C). Expression levels of mRNA for IL-10 in CCR8^-/-^ PMφ were constantly very low compared with those in WT PMφ. In contrast, the amount of IL-10 transcripts in BMMφ from CCR8^-/-^ mice was comparable to those from WT mice. As shown in Figure 1D, majority of the cells expressed CCR8 in both PMφ and BMMφ, although BMMφ showed higher intensity than PMφ. These results indicate that full induction of TNF-α and IL-6 in PMφ via TLR4 requires the presence of CCR8. IL-10 secretion via TLR4 and TLR1/2 was dependent on CCR8. These effects were more specific to PMφ than to BMMφ, this was not due to the surface expression levels of CCR8.

### Selection of CCR8 antagonist R243 using a CCL1/CCR8 functional assay

**Figure 2:**
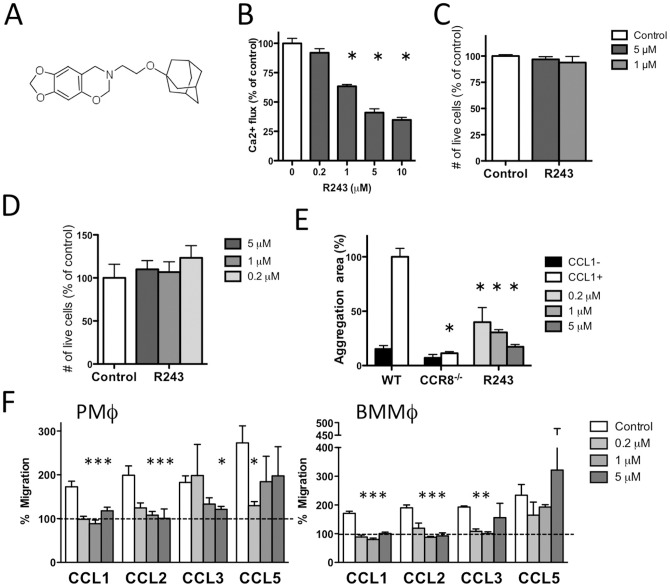
Small molecule R243 inhibits CCR8 signaling and chemotaxis. (A) Structure of R243. (B) R243 inhibition of CCL1-induced Ca^2+^ flux in CCR8-CHO cells. Fluorescence signal value is shown as percent of those cells treated with CCL1+0.1% DMSO (vehicle). Results are expressed as mean plus standard deviation (SD) from six assays for each condition. *Statistically significant difference from 0 (without R243) as determined by an unpaired two-tailed t test. (C) CCR8-CHO cells were cultured with 1 or 5 μM of R243 for 18 h, and the number of living cells was measured by WST assay. Results are expressed as mean plus SD of three assays. (D) PMφ were cultured for 24 h in the presence of indicated concentration of R243 and harvested with trypsin/EDTA. The numbers of living cells were counted using trypan-blue exclusion. Results are expressed as mean plus SD of three assays. (E) CIMA assay. WT or *CCR8^-/-^* PMφ were cultured on mesothelial cell monolayers with CCL1 for 24 h with various concentrations of R243. The aggregation area was measured and is shown as percent of that induced with CCL1 (CCL1+). Results are expressed as mean plus SD of five assays. *Statistically significant difference from CCL1-treated WT cells as determined by an unpaired two-tailed t test. (F) Chemotaxis of PMφ and BMMφ. Fluorescently labeled cells were stimulated with 10 ng/mL of CCL1, CCL2, CCL3, or CCL5, with or without R243 at the indicated concentrations. Migrated cells were quantified by fluorescent level and are shown as the percent of chemokine-induced migration to random migration ( = 100%). Results are presented as mean plus SD of four assays. *Statistically significant difference in migration between control and compound-treated cells as determined by an unpaired two-tailed t test.

Based on the result that CCR8 supported cytokine responses to inflammatory stimuli, we wished to obtain CCR8 antagonists. A low molecular weight compound, R243 (Fig. 2A), was initially selected as a possible CCR8 antagonist from a library (see Materials and Methods) based on its inhibitory activity on CCL1-induced Ca^2+^ flux in CCR8-expressing CHO cells (Fig 2B). Significant inhibition of CCL1-induced Ca^2+^ flux was observed with R243 at ≥1 μM. The presence of R243 did not affect cell viability at ≤5 μM after 18 h of culture in this CHO cell line (Fig 2C). Absence of cytotoxicity in mouse PMφ with R243 treatment at 5 μM was also confirmed (Fig. 2D). As a secondary *in vitro* biological screen for CCR8 antagonists, we used a CIMA assay, which detects CCL1/CCR8-specific interactions between mouse mesothelial cells and PMφ based on cell aggregation. R243 suppressed CCL1-induced cell aggregation in a dose-dependent manner (Fig. 2E). R243 suppressed aggregation at a concentration as low as 0.2 μM, and the inhibition of cell aggregation at a concentration of 5 μM was comparable to that seen for cells isolated from *CCR8^-/-^* mice. Our attempts to measure direct binding of R243 to CCR8 by ELISA or Biacore system analysis failed due to technical difficulties. High background from CCR8 immobilized to antibodies prevented sensitive detection of ligands in the ELISA. Determination of intact R243 binding to immobilized CCR8 failed due to very low levels of signal in the Biacore analysis. Thus, like many small molecule compounds reported as chemokine antagonists, binding to the receptor or the site of action for R243 remained undefined.

The specificity of R243 was investigated using a chemotaxis assay for PMφ and BMMφ (Fig. 2F). Naïve PMφ and BMMφ demonstrated chemotaxis in the presence of CCL1, CCL2, CCL3, and CCL5. R243 inhibited CCL1- and CCL2-induced chemotaxis in both types of macrophages; however, inhibition of CCL3-induced chemotaxis in PMφrequired a higher R243 concentration. A trend towards inhibition of CCL5-induced chemotaxis in PMφ was seen but R243 did not inhibit CCL5-induced chemotaxis of BMMφ. These results suggest that R243 inhibits chemotaxis via CCR8 and CCR2, a major receptor for CCL2, and potentially blocks CCR1 and CCR5, receptors for CCL3 and CCL5, respectively. Thus, R243 had inhibitory effects on CCL1/CCR8 with additional effects of inhibiting chemotaxis driven by some other chemokines.

### R243 reduces cytokine secretion by mouse PMφ and BMMφ

**Figure 3:**
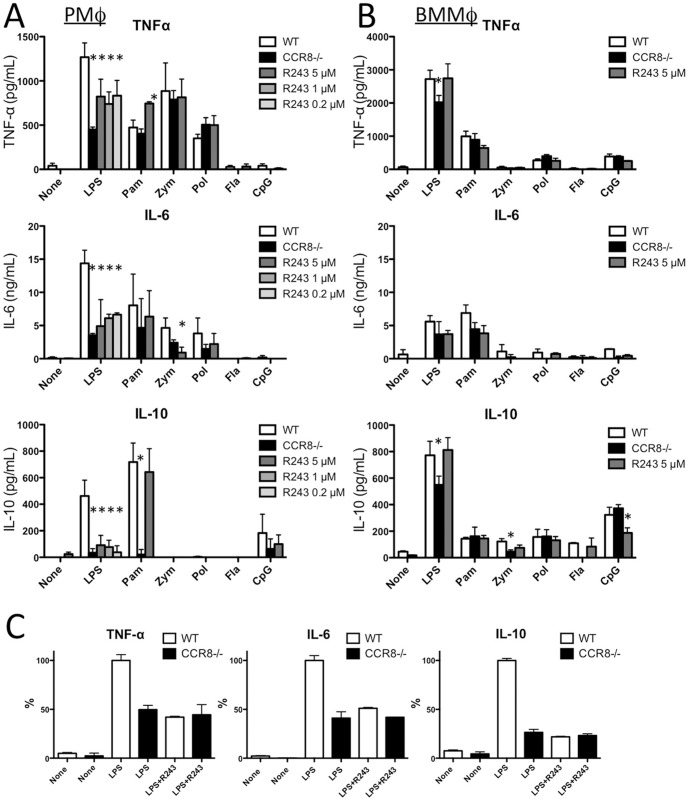
R243 inhibited LPS-induced cytokine production by mouse PMφ. (A) PMφ or (B) BMMφ were collected from naïve WT or *CCR8^-/-^* mice. WT cells were also treated with indicated concentration of R243 and cultured for cytokine assay as in Figure 1. (C) PMφ form naïve WT or *CCR8^-/-^* mice was stimulated with LPS as above in the presence or absence of R243 (5 μM) for cytokine assay. Results are shown as % of cytokine level of LPS-stimulated WT PMφ without R243. Results are presented as mean plus standard deviation (SD) of three assays. *Statistically significant difference between WT and *CCR8^-/-^* cells (Mann-Whitney test). In (C), there was no statistically significant difference among the values of LPS plus CCR8^-/-^ PMφ with R243, LPS plus CCR8^-/-^ PMφ without R243 and LPS plus WT PMφ with R243 in each cytokine assay.

Next, we examined the ability of R243 to inhibit TLR ligand-induced cytokine production in WT cells compared with *CCR8^-/-^* cells. Generally, the effects of R243 on WT PMφ were comparable to those seen in *CCR8* ^-/-^ PMφ except Pam3CSK4-induced IL-10, which was strongly suppressed in *CCR8* ^-/-^ PMφ but not affected with R243 treatment (Fig. 3A). The effect of R243 on BMMφ was relatively low when compared with the effect on PMφ. Mildly attenuated secretion of LPS-induced TNF-á and IL-10 observed in *CCR8* ^-/-^ BMMφ was not seen in WT PMφ treated with R243 (Fig 3B). When CCR8^-/-^ PMφ were stimulated with LPS in the presence of R243, R243 did not show any additional effect on CCR8 deficiency in terms of cytokines production in PMφ (Fig 3C). Thus, we found that R243 largely mimics the effect of CCR8 deficiency, suggesting that R243 inhibits LPS-induced cytokine production via antagonism of CCR8. To confirm the biological effect of commercially obtained R243, we synthesized a compound with the same structure as R243. Our synthetic compound demonstrated exactly the same suppressive effects in CIMA and LPS-triggered cytokine production assays.

### Intracellular localization of LPS and TLR was not affected in *CCR8^-/-^* or R243-treated PMφ

**Figure 4:**
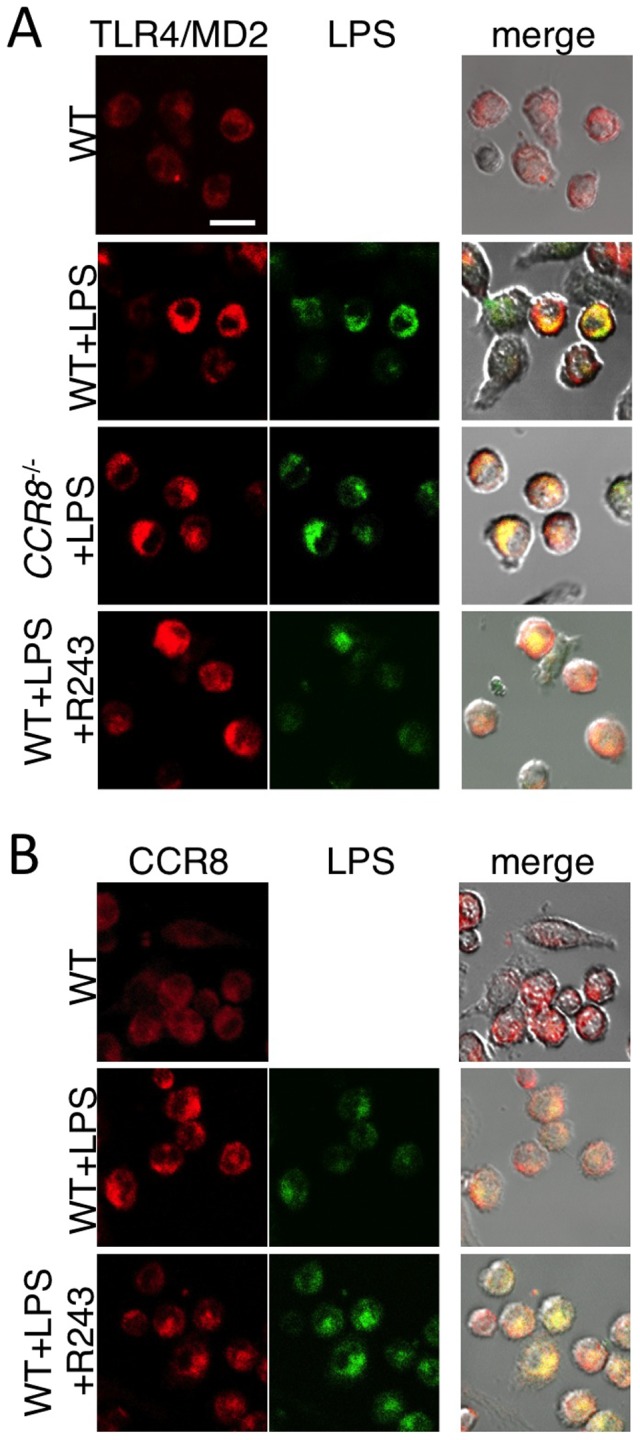
Localization of TLR4 and LPS in WT, *CCR8^-/-^*, and R243-treated PMφ. (A) WT PMφ, *CCR8^-/-^* PMφ or WT PMφ in the presence of R243 (5 μM) were stimulated with or without FITC-conjugated LPS (1 mg/mL) for 45 min and stained with biotin-conjugated anti-TLR4/MD2 and TRITC (red)-streptavidin. (B) WT PMφ were stimulated with FITC (green)-conjugated LPS, as in panel A, with or without R243 (5 μM) and stained with anti-CCR8 antibody and anti-sheep IgG-conjugated with Texas Red (red). Fluorescent images were merged and overlaid on the differential interference contrasted image. Bar = 20 μm.

We further investigated the mechanism of the CCR8-dependent LPS-response using R243-treated and *CCR8* ^-/-^ cells in parallel. We examined whether *CCR8* deficiency or R243 treatment affected internalization of the LPS-TLR4 complex. Consistent with the previous reports [21], [22], LPS triggered TLR4 clustering co-localized with LPS in the intracellular compartment (Fig. 4A). We found that CCR8 also localized together with LPS after LPS stimulus (Fig. 4B). However, there was no difference in the intracellular localization of TLR4 and fluorescent-labeled LPS between WT, R243-treated, or *CCR8^-/-^* PMφ (Fig. 4A). R243 treatment did not alter the expression level of CCR8 or distribution of LPS (Fig. 4B). Thus, neither the absence of CCR8 nor presence of R243 significantly affected the recognition and trafficking of LPS by TLR4.

### Neutralization of CCL1 did not affect LPS-induced IL-10 secretion

**Figure 5:**
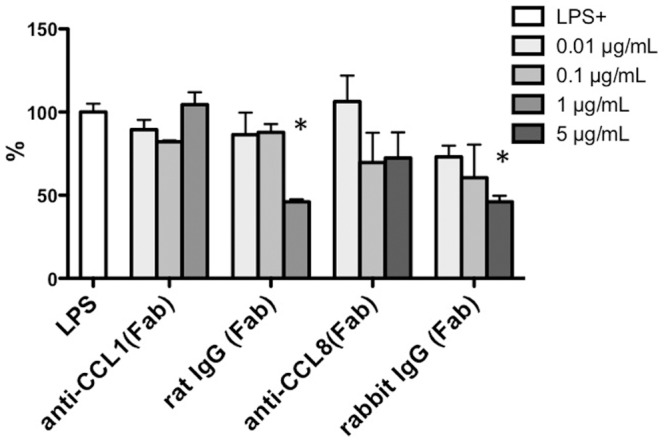
CCR8 ligand secretion was not required for LPS-induced IL-10 secretion. WT PMφ were stimulated with LPS (100 ng/mL) in the presence of the indicated concentrations of anti-CCL1, anti-CCL8, or control IgG (rat and rabbit, respectively, all F(ab) fragments) for 24 h. IL-10 in the culture supernatant was then measured. Data are shown as the percent of the level in the culture medium of cells stimulated only with LPS. Results are expressed as the mean plus standard deviation (SD) of two or three assays. *Statistically significant difference from LPS only cultures (Mann-Whitney test).

Because we previously found that LPS upregulates CCL1 transcription in PMφ[14], we also examined the requirement of autocrine/paracrine CCL1 secretion for LPS-induced cytokine production by using the F(ab) fragments of anti-CCL1 or anti-CCL8 antibodies to capture endogenous, extracellular CCR8 ligands. This treatment did not affect LPS-induced production of IL-10 (Fig. 5), although a non-specific effect of the F(ab) fragment from control rat IgG and rabbit IgG was seen at the high concentration of 5 μg/ml. Anti-CCL1 Fab did not affected the production of TNF-α or IL-6, either. These results suggested that IL-10 production depends on the presence of CCR8, but that CCR8 activation by chemokine ligand binding may not be necessary.

### Intracellular signaling in *CCR8^-/-^* PMφ and R243-treated PMφ

**Figure 6:**
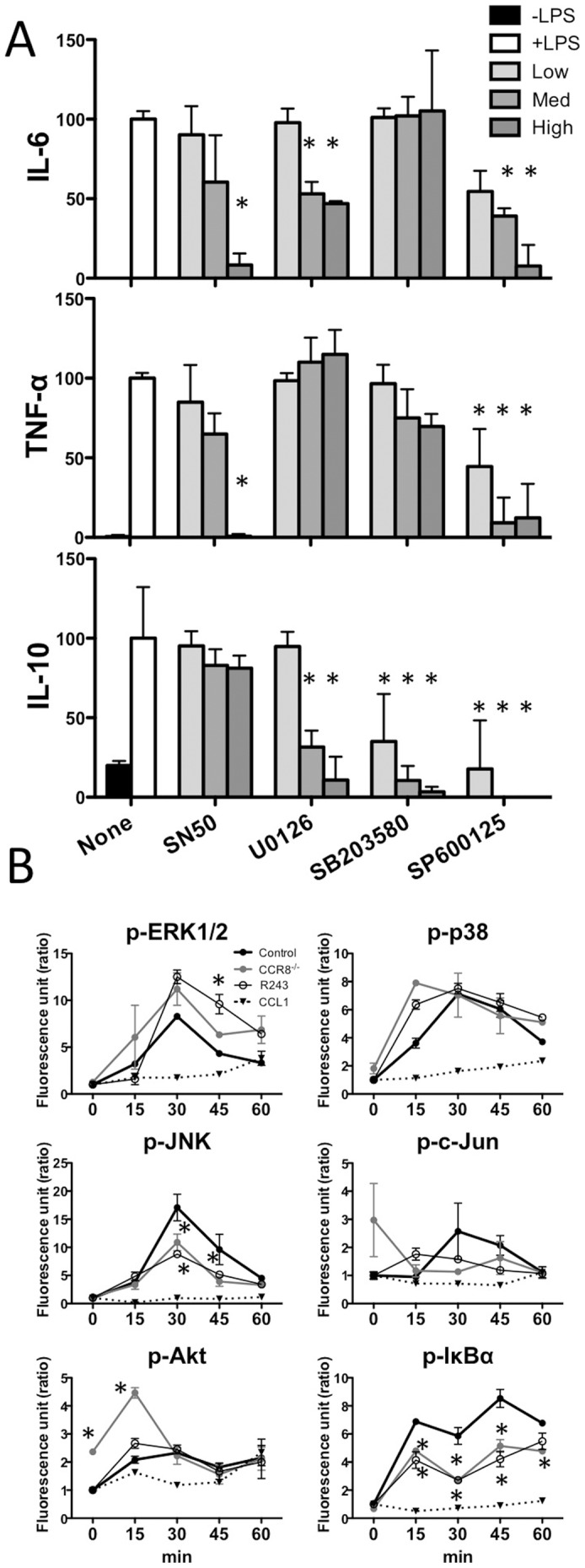
Target of CCR8 signaling and R243 in LPS-induced cytokine production by PMφ. (A) WT PMφ were stimulated with LPS (100 ng/mL) for 24 h in the absence (none) or presence (low, medium, and high indicate 0.5, 5, and 50 μM, respectively) of SN50, U0120 (0.1, 1, 10 μM), SB203580 (0.1, 1, 10 μM), or SP600125 (0.5, 5, 50 μM). The levels of cytokines in the culture medium were measured and are shown as percent relative to the LPS-stimulated cells. *Statistically significant difference between cells treated with LPS plus inhibitors and cells treated with LPS only (P<0.05, Mann-Whitney test). (B) WT (control), *CCR8^-/-^* PMφ or WT PMφ treated with R243 were stimulated with LPS (100 ng/mL) for the indicated times. Some cells were stimulated with 100 ng/ml of CCL1 without LPS. Each phosphoprotein was quantified using a multiplex phosphoprotein detection assay. The intensity relative to unstimulated WT PMφ (0 min) results are expressed as the mean with standard deviation (SD) of three assays. *Statistically significant difference compared with control (two-way ANOVA with Bonferroni post-tests).

To identify the pathway responsible for CCR8-dependent cytokine production, we examined the effect of various signaling inhibitors. Treatment of LPS-stimulated PMφwith the NF-κB inhibitor SN50 significantly reduced production of IL-6 and TNF-α but did not affect IL-10 production (Fig. 6A). Treatment with the ERK inhibitor U0126 resulted in moderate reductions in IL-6 and IL-10 secretion but not TNF-α secretion (Fig. 6A). The p38 inhibitor SB203580 had no effect on IL-6 or TNF-α levels but significantly attenuated IL-10 secretion, whereas the JNK inhibitor SP600125 reduced secretion of all cytokines (Fig. 6A). These results indicate that IL-10 secretion involves the MAP and JNK pathways rather than NF-κB. The inhibitors of JNK induced an effect similar to that seen in *CCR8^-/-^* and R243-treated cells.

Next, we determined the phosphorylation state of ERK, p38, JNK, c-Jun, Akt, and IκBα using a phosphoprotein detection assay (Fig. 6B). In R243-treated and *CCR8^-/-^* PMφ, the phosphorylation levels of JNK and IκBα were lower than in WT PMφ. Phospho (p)-c-Jun also showed a trend of decreased phosphorylation. Phosphorylation of ERK, Akt, and p38 was not suppressed by R243 treatment or *CCR8* deficiency. In this experiment, the effects of CCR8 deficiency and R243 treatment were similar, except for high Akt phosphorylation in unstimulated *CCR8^-/-^* PMφ and 15 min after LPS-stimulation. In contrast to LPS, CCL1 did not show significant activation of any of these signaling pathways. Together with the results of inhibitor assays, these results indicate that both CCR8 deficiency and R243 treatment suppress IL-10 production by inhibiting the MAPK/JNK/AP-1 signaling pathway. CCR8 deficiency and R243 also partially attenuated the NF-κB pathway, resulting in reduced production of IL-6 and TNF-α and this function of CCR8 may not be directly induced by CCL1 binding and independent from CCL1 triggered chemotaxis/cell aggregation.

### Effect of CCR8 deficiency and R243 in *in vivo* inflammation models

**Figure 7:**
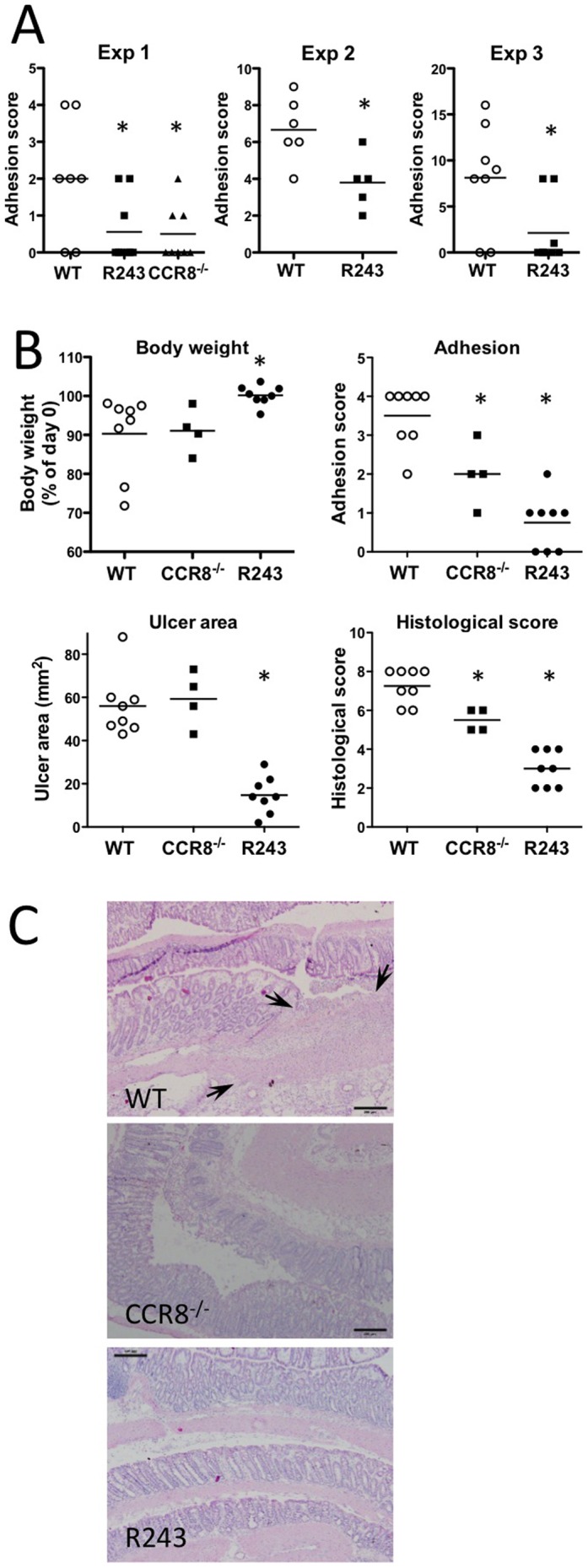
*CCR8* deficiency and R243 attenuated postoperative peritoneal adhesions and acute colitis *in vivo*. WT, *CCR8^-/-^*, or WT mice administered with R243 were compared. (A) Mouse peritoneal adhesions were induced through formation of peritoneal ischemic buttons (Exp 1, single R243 dose on day 0), by ablation of the cecum with an electric scalpel (Exp 2, three R243 doses on days 0, 2, and 4), and laparotomy and abrasion of the cecum with gauze (Exp 3, single R243 dose on day 0). Each dot represents an individual mouse. The short bar indicates mean value. *Statistically significant difference compared with the control (WT) according to an unpaired two-tailed t test. (B) Colitis was induced by intrarectal administration of TNBS. R243 was injected intraperitoneally on days 0 and 2. Other mice were given vehicle solution. Each dot represents an individual mouse. The short bar indicates a mean value. *Statistically significant difference from WT control (Mann-Whitney test). (C) Typical histological findings of colitis. Frozen sections were stained with hematoxylin and eosin. Bar = 200 μm. Arrows indicate transmural lesion with peritoneal adhesions.

Because we found that R243 treatment and CCR8 deficiency impact inflammatory responses, we evaluated their effect in disease models. A mouse peritoneal adhesion model was used to investigate *in vivo* inhibition of CCL1/CCR8 by R243 because the preventive effect of anti-CCL1 antibody and CCR8 deficiency have already been established [14]. Three different methods for inducing peritoneal adhesions were examined, including formation of ischemic buttons, ablation of the cecum with an electric scalpel, and abrasion of the cecum with gauze. In all models, administration of R243 prevented postoperative adhesions (Fig 7A). This result indicates that R243 efficiently suppresses the CCL1/CCR8 pathway.

In contrast to a sterile adhesion model using ischemic buttons, adhesions induced with cecal ablation or cecal abrasion involves stimuli from bacterial component in the cecum. The effect of R243 treatment in these models, in addition to LPS-triggered cytokine production, prompted us to examine its effect on acute colitis. Acute colitis was induced by intrarectal injection of the hapten trinitrobenzene sulfonic acid (TNBS) on day 0. On day 4, mice developed necrotizing focal ulcers in the distal colon (Fig. 7B and 7C). Colitis-associated inflammation in *CCR8* ^-/-^ mice was less severe than that in WT mice, as evidenced by less peritoneal adhesion to the colon and lower histological scores indicating fewer transmural ulcers, but there were no significant decreases in ulcer area (Fig. 7B). In contrast, two intraperitoneal injections of R243 on days 0 and 2 significantly ameliorated the acute colitis, as evidenced by a decrease in body weight loss, less adhesion, reduced open ulcer area, and fewer histological changes (Figs. 7B and 7C).

## Discussion

The mechanism of macrophage activation by TLR agonists has been a central focus of innate immunity research and has been studied extensively in recent decades using mouse PMφ. Here, we present the first report that CCR8 plays an essential role in LPS-induced cytokine production In particular, IL-10 secretion by PMφ is almost completely dependent on CCR8. The small-molecule compound R243, which was identified based on its inhibition of CCL1/CCR8 interaction, produced the same effect on IL-10 secretion as did CCR8 deficiency.

**Figure 8:**
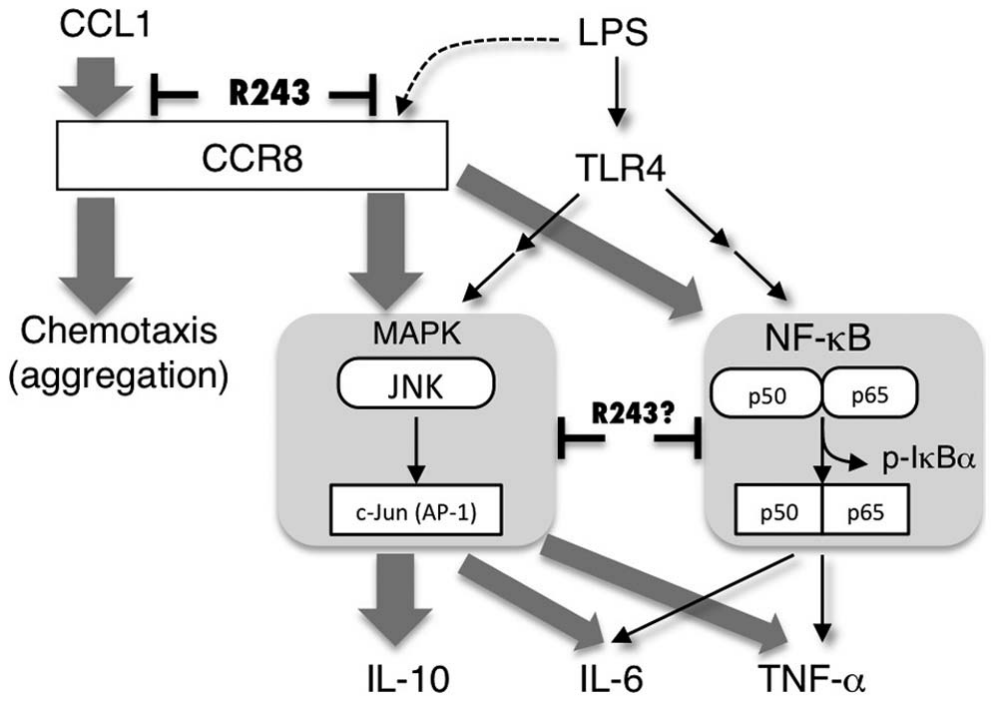
Scheme of TLR4 and CCR8 signaling cross talk in mouse PMφ. LPS triggers TLR4 signaling and induces NF-κB and MAPK activation, possibly together with CCR8 activation, which results in the production of TNF-α, IL-6, and IL-10. CCR8 is required for full activation of both signaling pathways. Production of IL-6 and TNF-α involves both the NF-κB and MAPK pathways. IL-10 production is largely dependent on the JNK pathway and is mediated by CCR8 activation. In addition to the antagonistic effect on CCR8, possible sites of action of R243 are indicated.

Our major finding was that of cross talk between CCR8 and the TLR4 signaling pathway. CCR8-dependency in LPS-response was not simply due to the surface expression levels of CCR8, but to the intracellular signaling system. This system functions particularly in PMφ as terminally differentiated tissue macrophages, but not in *ex vivo* induced BMMφ. TLR4 signaling triggers both MyD88- and TRIF-dependent pathways. Although TRIF is an adaptor molecule for TLR3 and induces TNF-α secretion by PMφ, the TLR3 pathway was not affected by either CCR8 deficiency or R243 treatment. In addition, IL-10 secretion was not induced by the TLR3 ligand in PMφ. These results indicate that the interaction between CCR8 and the TLR4 pathway is not TRIF-dependent, but is MyD88-dependent. Downstream of MyD88, activation of NF-κB and MAPK (including ERK, JNK, and p38)-AP1 occurs, eventually facilitating transcription of inflammatory cytokines. Based on the results of inhibitor experiments and the observed decreases in p-JNK, p-c-Jun, and p- IκBα in LPS-stimulated *CCR8^-/-^* PMφ relative to WT PMφ, we conclude that LPS-induced activation of both NF-κB and JNK signaling involves CCR8. R243 treatment produced the same pattern of signaling pathway modulation as was observed in *CCR8^-/-^* PMφ. Secretion of IL-10 is mediated almost entirely by activation of JNK rather than NF-κB, and dependence of JNK signaling on CCR8 would disrupt LPS-induced IL-10 production in *CCR8^-/-^* or R243-treated cells. Production of IL-6 and TNF-α involves both NF-κB and JNK pathways, which are probably partially dependent on the presence of CCR8. The possible pathways are illustrated in Figure 8.

The results of previous paper [23], indicating that CCR8^-/-^ mice are resistant to the endotoxemia induced by cecal ligation and puncture and display decreased plasma levels of TNF-α, are consistent with our result that cecal abrasion induced fewer pathological inflammatory lesions in CCR8^-/-^ mice than in WT mice. However, they found augmented secretion of TNF-α in LPS-treated CCR8^-/-^ peritoneal adherent cells, in contrast to our finding of attenuated secretion of TNF-α in CCR8^-/-^ peritoneal macrophages. The reason for this difference is not clear, but it is possibly due to the different methods used for cell preparation and different culture conditions. They used culture medium supplemented with 10% serum for both cell adhesion and stimulation with 1 μg/ml of LPS, whereas we used 1% serum in all experiments and 100 ng/ml of LPS for the cytokine assay. The 10-fold higher concentration of serum and LPS might have caused the cell composition of the adherent cell fraction to be different from that in our study. This difference in concentration may also have influenced LPS signaling because of the abundance of LPS, LPS-binding protein, or CD14. Nonetheless, we think both reports consistently indicate that CCR8 enhances the overall pathological signaling via TLR4.

How CCR8 supports TLR4 downstream signaling remains unclear. Although details of the interaction between CCR8 and TLR4 are limited, cross talk between GPCRs and TLRs was suggested in the 1980's [24], [25]. Chemokine receptors belong to a large family of GPCRs, the rhodopsin-like family, and their activation induces G-protein coupling, which is abrogated by pertussis toxin (Ptx). Several lines of evidence suggest that PTx-sensitive G-proteins regulate not only GPCR signaling but also TLR signaling in macrophages [26]. Pretreatment of macrophages with PTx was initially reported to reduce IL-1 production in response to LPS [25]. In human monocytic U937 cells, PTx also impairs LPS-induced IL-1 responses and Gαi2 phosphorylation, implicating GPCR involvement in LPS signaling [24]. In mouse thioglycollate-elicited PMφ, PTx has been shown to inhibit LPS-dependent NO production but to amplify TNF production [27], although others reported that PTx has no effect on TNF secretion [28]. The GPCR chemokine receptor CXCR4 was identified as an LPS receptor cluster protein using peptide mass fingerprinting [29]. Overall, these reports suggest that activation of GPCRs, including chemokine receptors, augments LPS signaling, although the signaling pathway may vary depending on cell type and activation status. Our finding that LPS-triggered cytokine production does not require free CCL1 or CCL8 suggests that CCR8 is activated endogenously, without new ligand ligation, upon LPS stimulation. LPS upregulates CCR8 expression and induces clustering in the PMφsurface membrane [14], and this may be associated with endogenous activation of CCR8. Furthermore, inflammatory activation of PMφ downregulates many chemokine receptors expressed in naïve cells, except CCR8 [14], and this may alter the localization or conformation of CCR8 in the plasma membrane, thereby enhancing TLR4 signaling. Although we saw no microscopic change in LPS/TLR4 internalization in *CCR8^-/-^* or R243-treated PMφ, interaction between TLR4 and CCR8 at the molecular level remains to be investigated.

In spite of the broad specificity of R243, we used it as a CCR8 antagonist in this study, because once PMφ are activated with LPS or CCL1, CCR8 is primarily a single chemokine receptor, as we previously reported [14]. Expression of other chemokine receptors are strikingly downregulated and their functions are likely minimal. As a consequence of this unique feature of peritoneal macrophages, the major action of R243 should be focused on CCR8 in LPS-stimulated peritoneal macrophages. Whether R243 inhibits ligand binding to CCR8 on the cell surface or modulates CCR8 function by acting on downstream signaling molecules remains undetermined. However, our results suggest that R243 affects TLR4-triggered signaling molecules such as JNK, c-Jun, and IκBα but does not affect ERK1/2 and p38. Furthermore, R243 shows a TLR preference. For example, TLR2-triggered TNF-α production is also mediated by MyD88 activation, but is minimally reduced with R243 treatment or *CCR8* deficiency. We believe it is unlikely that a small molecule, such as R243, interacts directly with multiple target molecules in each pathway. It may be possible that R243 nonspecifically modulates G-protein function, but failure to block BMMφ CCL5-driven chemotaxis suggests that R243 has a degree of receptor specificity. Thus, R243 most likely mimics CCR8 deficiency by inhibiting activation of CCR8. In this sense, the phenotypes of CCR8 deficiency and R243 treatment are mostly compatible. However, our results indicate there are two differences: 1) Pam3CSK4-induced IL-10 production is abrogated in *CCR8^-/-^* PMφ but not with R243 treatment, and 2) suppression of TNBS colitis in *CCR8^-/-^* mice is not as evident as that in R243-treated mice. Pam3CSK4 is recognized by TLR1/2, which may be in a condition distinct from that of TLR4 in the sense of total CCR8 protein deficiency and interaction with R243-modulated CCR8. In the case of colitis, the potential for R243 to block both CCL2 (MCP-1)/CCR2-driven chemotaxis and CCL1 might enhance the anti-inflammatory effect in comparison with that of *CCR8^-/-^* mice. Indeed, in the colitis model, activation of the CCL1/CCR8 system is limited to the serosal side of the colon or the peritoneal cavity [14]. In contrast, significance of CCL2/CCR2 in colitis models has been established [30]–[33]. CCL2 is abundant and plays a major role in recruiting hematogenic BMMφ as well as T cells and dendritic cells, to inflamed sites in the colonic mucosa. The potential of R243 to suppress the function of multiple chemokines might result in a more efficient anti-inflammatory response than that seen in *CCR8^-/-^* mice. Although R243 treatment reduced IL-10 secretion, its overall suppression of TNF-α and IL-6, together with inhibition of inflammatory cell infiltration, seemed to ameliorate colitis.

In conclusion, our results demonstrated that CCR8 was significantly involved in the TLR4-mediated signaling pathway. In addition, R243 and compounds with R243-related structure may serve as seeds for new drug development research.

## Supporting Information

Method S1 (DOCX)
