# Supplementary material for: Chemokine Receptor CCR8 Is Required for Lipopolysaccharide-Triggered Cytokine Production in Mouse Peritoneal Macrophages
Source: PLoS One. 2014 Apr 8;9(4):e94445. doi: 10.1371/journal.pone.0094445 (PMC3979852; doi:10.1371/journal.pone.0094445)
Supplement: Method S1 — (DOCX) [file pone.0094445.s001.docx]

**Method S1**

**Supporting information for**

**Chemokine receptor CCR8 is required for lipopolysaccharide-triggered cytokine production in mouse peritoneal macrophages**

**Materials and Methods**

**Reagents.** Recombinant mouse CCL1/TCA-3, CCL2/JE/MCP-1, CCL3/MIP-1α, and CCL5/RANTES were purchased from R&D Systems (Minneapolis, MN). For TLR agonists, LPS from *E. coli,* FITC-conjugated LPS from *E. coli* (L3024), and flagellin were purchased from Sigma-Aldrich (Tokyo, Japan). Zymosan, Pam3CSK4, and PolyI:C were purchased from IMGENEX (San Diego, CA). CpG-ODN was purchased from Takara Bio Inc. (Shiga, Japan). Inhibitors SB203580 (p38 inhibitor), SP600125 (JNK inhibitor), U0126 (ERK inhibitor), and SN50 (NF-κB inhibitor) were purchased from Calbiochem (La Jolla, CA). The following antibodies were used: rat anti-mouse CCL1/TCA-3, rat IgG2b (both from R&D systems), rabbit anti-CCL8/MCP-2 (Leinco Technologies Inc., St. Louis, MO), normal rabbit IgG (Santa Cruz Biotechnology, Inc. Dallas, TX), sheep anti-mouse/rat CCR8 (R&D Systems), TRITC-conjugated streptavidin (Southern Biotech, Birmingham, AL), Texas Red-conjugated anti-sheep IgG (Santa Cruz Biotechnology), and Texas Red-conjugated anti-sheep IgG (Santa Cruz Biotechnology). Biotin-anti-mouse TLR4/MD2 antibody (SA15-21) was kindly provided by Dr. S. Takamura-Akashi (Tokyo University). For blocking experiments, F(ab) fragments of anti-CCL1, anti-CCL8, and control IgG were prepared using a Pierce Fab Micro Preparation Kit (Thermo Scientific, Tokyo, Japan). Blocking activity of F(ab) antibodies was confirmed based on inhibition of CCL1-induced Ca^2+^ flux in CCR8-expressing CHO cells.

**ELISA for IL-6, IL-10, and TNF-α** IL-6, IL-10, and TNF-α concentrations in culture supernatants were measured using an ELISA development kit (Peprotech) according to the manufacturer’s instructions.

**Total RNA purification, gene profiling and Quantitative RT-PCR (qRT-PR).** Total RNA was extracted and purified using AllPrep Micro Kit (Qiagen, Tokyo, Japan). After reverse transcription, expression level of each gene was quantified using TaqMan gene expression kit (for TNF-α and 18s rRNA) (Applied Biosystems, Warrington, England) or a SYBR Green PCR Master Mix (IL-10 and IL-6) with ABI PRISM 7900 Sequence Detector (Applied Biosystems). Primers used were as follows; mouse IL-6, 5’-TGGAGTCACAGAAGGAGTGGCTAAG and 5’-TCTGACCACAGTGAGGAATGTCCAC, mouse IL-10, 5’-ACCTGGTAGAAGTGATGCCCCAGGC and 5’-CTATGCAGTTGATGAAGATGTCAAA. The step-cycle program was annealing at 60°C for 45 seconds and extension at 72°C for 45 seconds for a total of 40 cycles. Threshold cycle numbers (Ct) were determined with Sequence Detector Software (version 1.7; Applied Biosystems) and transformed by using the ΔCt/ΔΔCt method as described by the manufacturer, with 18s rRNA used as the calibrator gene.

**Flow cytometory.** Cells were stained with PE-labeled anti-F4/80 ([BD Biosciences](http://www.bdbiosciences.com/instruments/facscalibur/)) and goat anti-CCR8 antibody (Abcam, Tokyo, Japan) followed by FITC-labeld anti-goat IgG antibody (Southern Biotech), and analylzed with FACS Calibur ([BD Biosciences](http://www.bdbiosciences.com/instruments/facscalibur/)).

**Living cell counting.** Cells were inoculated in 96-well plates at the concentration of 2 × 10^3^/well with indicated concentration of R243 or 0.1% DMSO (vehicle solution for R243). Cells were cultured for 18 h, and then 10 μL of Tetracolor (WST-8 solution, SEIKAGAKU Co., Tokyo Japan) was added to each well. After 2 h of additional culture, the number of living cells was measured as absorbance at 450 nm.

**Chemotaxis assay.** Aliquots of PMφ or BMMφ cultures were prestained for 30 min at 37°C with 3 μg/mL of BCECF-AM (Molecular Probes) and then suspended at 1 × 10^6^ cells/mL in DMEM containing 0.5% BSA and 20 mM HEPES. Chemotaxis assays were performed using a Chemo Tx-96 Chemotaxis Plate (NeuroProbe) as follows: 65 μL of cell suspension with serially diluted R243 was loaded onto the membrane plate, and the plate was placed into a well in a flat-bottom microtiter plate containing 30 μL of recombinant chemokine ligand solution (10 ng/mL). The plate was then incubated at 37°C for 90 min, and the fluorescence intensity of cells that migrated in the wells was measured using a microplate reader (FlexsStation 3, Molecular Devices, Tokyo, Japan).

**Immunofluorescence test.** WT or *CCR8^-/-^* PMφ were seeded in a glass-base 60-mm culture dish (2 × 10^5^ cells/well) and incubated with biotin-anti-TLR4/MD2 or anti-CCR8 antibody for 60 min followed by TRITC-conjugated streptavidin or Texas Red-conjugated anti-sheep IgG for 60 min, respectively., then stimulated with FITC-conjugated LPS for 45 min at 37°C in the presence or absence of R243. Cells were washed three times with PBS and then fixed with 4% paraformaldehyde/PBS for 15 min, and then blocked with Block Ace in PBS or 10% normal rabbit serum (HISTOFINE, Nichirei Biosciences Inc., Tokyo, Japan) for 30 min at room temperature. Antibodies were detected by Images were obtained by confocal fluorescence microscopy (Olympus FV1000, Tokyo, Japan).

**Phosphoprotein assays.** WT, *CCR8^-/-^* PMφ, or WT PMφ with 50 μM R243 were seeded in 96-well plates (3 × 10^5^ cells/well) and then stimulated with 100 ng/mL of LPS at 37°C for 0–60 min. Cell lysates were prepared using a Bio-Plex Cell Lysis kit (BIO-RAD, Hercules, CA). Lysates were analyzed for quantification of p-ERK, p-p38, p-JNK, p-Akt, p-c-Jun, and p-IκBα using a Bio-Plex Phospho 6-plex assay kit (BIO-RAD) or Bio-Plex Total 6-plex assay kit (BIO-RAD) according to the manufacturer’s instructions.

**Mouse models of peritoneal adhesion.** Three different methods were used to induce peritoneal adhesions in mice. In method 1, after laparotomy, ischemic buttons were formed in the peritoneal wall by grasping the peritoneum with a hemostat clamp and ligating the base of the segment with a 4-0 silk suture. In method 2, laparotomy was followed by ablation of the cecum using an X-10 EM-1001 electric scalpel (Bovie Medical Corp., Clearwater, FL). In method 3, laparotomy was followed by abrasion of the cecum with dry gauze until redness appeared. In all experiments, mice were anesthetized with ketamine plus xylazine. Adhesions were assessed on day 6.

**Scoring systems for *in vivo* models.** The adhesion score for ischemic buttons was determined as follows: 0 = no adhesion; 1 = thin filmy adhesion; 2 = thick planar adhesion. Scores were multiplied by the number of adhesions and summed for 2 buttons/mouse. Scores for cecal ablation or abrasion were as follows and were multiplied by the number of adhesions: 1 = weak (removable without cutting) adhesion; 2 = tight (need to cut to remove adhesion) or weak adhesion of 5–10 mm in length; 3 = weak adhesion of 10–20 mm in length; 4 = tight adhesion of 5–10 mm in length; 5 = tight adhesion of 10-20 mm in length. Scores for adhesion onto the colonic surface in TNBS colitis were as follows: 0, no adhesion; 1, one thin filmy adhesion; 2, more than one thin adhesion; 3, thin adhesion with focal point: 4, thick adhesion with plantar attachment or more than one thick adhesion with focal point; 5, very thick vascularized adhesions with more than one plantar attachment.

Clinical scores for colitis were as follows: 0 = normal stool; 1 = soft stool; 2 = diarrhea or proctitis; 3 = anal bleeding; 4 = dead or moribund. Ulcer area was determined from captured macroscopic images using Image J software (NIH). The histological score for colitis was as follows (values from the proximal, middle, and distal colon were summed): 0 = normal; 1 = ulcer or cell infiltration limited to the mucosa; 2 = ulcer or limited cell infiltration in the submucosa; 3 = focal ulcer involving all layers of the colon; 4 = multiple lesions involving all layers of the colon or necrotizing ulcer 3 mm in length.
